# Long-Term Outcomes of Bovine versus Porcine Mitral Valve Replacement: A Multicenter Analysis

**DOI:** 10.1155/2023/2111843

**Published:** 2023-06-30

**Authors:** M. Broadwin, N. Ramkumar, D. J. Malenka, R. D. Quinn, C. S. Ross, F. Hirashima, J. D. Klemperer, R. S. Kramer, G. L. Sardella, B. Westbrook, A. W. Discipio, A. Iribarne, M. P. Robich

**Affiliations:** ^1^Department of Surgery, Lehigh Valley Health Network, 1200 South Ceder Crest Blvd, PA 18103, Allentown, USA; ^2^Geisel School of Medicine at Dartmouth College, 1 Rope Ferry Road, NH 03755, Hanover, USA; ^3^Department of Medicine, Section of Cardiovascular Medicine, Dartmouth-Hitchcock Medical Center, NH 03756, Lebanon, USA; ^4^Department of Surgery, Cardiovascular Institute, Maine Medical Center, 22 Bramhall Street, ME 04102, Portland, USA; ^5^Department of Surgery, Section of Cardiac Surgery, University of Vermont Medical Center, 111 Colchester Avenue, VT 05401, Burlington, USA; ^6^Northern Light Cardiology, Northern Light Eastern Maine Medical Center, 417 State Street, ME 04401, Bangor, USA; ^7^Department of Surgery, Catholic Medical Center, 100 McGregor Street, NH 03102, Manchester, USA; ^8^Department of Surgery, Section of Cardiac Surgery, Dartmouth-Hitchcock Medical Center, 1 Medical Center Drive, NH 03756, Lebanon, USA; ^9^Department of Surgery, Cardiothoracic Surgery, Staten Island University Hospital at Northwell Health, 475 Seaview Avenue, NY 10305, Staten Island, USA; ^10^Johns Hopkins Hospital, Division of Cardiac Surgery, 1800 Orleans St, Zayed 7107, MD 21287, Baltimore, USA

## Abstract

**Introduction:**

Recent national guidelines recommending mitral valve replacement (MVR) for severe secondary mitral regurgitation have resulted in an increased utilization of mitral bioprosthesis. There is a paucity of data on how longitudinal clinical outcomes vary by prosthesis type. We examined long-term survival and risk of reoperation between patients having bovine vs. porcine MVR. *Study Design*. A retrospective analysis of MVR or MVR + coronary artery bypass graft (CABG) from 2001 to 2017 among seven hospitals reporting to a prospectively maintained clinical registry was conducted. The analytic cohort included 1,284 patients undergoing MVR (801 bovine and 483 porcine). Baseline comorbidities were balanced using 1 : 1 propensity score matching with 432 patients in each group. The primary end point was all-cause mortality. Secondary end points included in-hospital morbidity, 30-day mortality, length of stay, and risk of reoperation.

**Results:**

In the overall cohort, patients receiving porcine valves were more likely to have diabetes (19% bovine vs. 29% porcine; *p* < 0.001), COPD (20% bovine vs. 27% porcine; *p*=0.008), dialysis or creatinine >2 mg/dL (4% bovine vs. 7% porcine; *p*=0.03), and coronary artery disease (65% bovine vs. 77% porcine; *p* < 0.001). There was no difference in stroke, acute kidney injury, mediastinitis, pneumonia, length of stay, in-hospital morbidity, or 30-day mortality. In the overall cohort, there was a difference in long-term survival (porcine HR 1.17 (95% CI: 1.00–1.37; *p*=050)). However, there was no difference in reoperation (porcine HR 0.56 (95% CI: 0.23–1.32; *p*=0.185)). In the propensity-matched cohort, patients were matched on all baseline characteristics. There was no difference in postoperative complications or in-hospital morbidity and 30-day mortality. After 1 : 1 propensity score matching, there was no difference in long-term survival (porcine HR 0.97 (95% CI: 0.81–1.17; *p*=0.756)) or risk of reoperation (porcine HR 0.54 (95% CI: 0.20–1.47; *p*=0.225)).

**Conclusions:**

In this multicenter analysis of patients undergoing bioprosthetic MVR, there was no difference in perioperative complications and risk of reoperation of long-term survival after matching.

## 1. Introduction

Recent national guidelines recommending mitral valve replacement (MVR) for severe secondary mitral regurgitation have resulted in an increased utilization of mitral bioprosthetic valves. Similarly, a decline in the use of mechanical mitral valves has seen a compensatory increase in the use of bioprosthetic valves [1]. Given that bioprosthetic valves do not require lifelong anticoagulation, they have become an increasingly attractive option in the treatment of valvular heart disease. Currently, the two most popular bioprosthetics on the market are the porcine Moasic (Medtronic, Minneapolis, MN) valve and the bovine pericardial Perimount (Edwards Lifescience, Irving, CA) valve. There has been a significant investigation into the performance of various brands of bioprosthetic valves in the aortic position; however, in the mitral position, there still exists a paucity of data on how longitudinal clinical outcomes vary by prosthesis types [2–7].

Previous studies on the use of bioprosthetic valves in the aortic position seem to favor the durability of bovine pericardial valves over that of porcine. Additional studies call into question the durability of bioprosthetics in younger patients [8, 9]. Surprisingly, investigations comparing the two bioprosthetic valves in the mitral position have been contradictory, with some data suggesting a possible longitudinal structural advantage to porcine valves in the mitral position and others favoring outcomes of bovine pericardial MVR; however, these studies were limited by either small sample size, limited number of surgeons, or were performed at a single institution [10, 11].

Given this overall paucity of data and lack of heterogeneity in study populations, comparing outcomes of bioprosthetic valves in the mitral position is difficult. Furthermore, given the increased risk of structural valve deterioration (SVD) in bioprosthetic mitral valves when compared with valves in the aortic position, long-term data about MVR is especially important. The purpose of this study was to examine long-term survival and risk of reoperation between patients having bovine vs. porcine MVR.

## 2. Methods

### 2.1. Data Source

We examined data from the Northern New England Cardiovascular Disease Study Group (NNECDSG). The NNECDSG is a multicenter, voluntary, regional collaboration of interventional cardiology, cardiac surgery, and structural heart programs in northern New England. The collaboration started in 1987 with a mission to improve the outcomes of cardiac interventions. Members from the seven medical centers include surgeons, cardiologists, anesthesiologists, perfusionists, nurses, other providers, administrators, and researchers. Institutional review boards (IRBs) at all the medical centers have designated the NNECDSG as a quality improvement registry, and therefore, patient consent for data collection was not required. Registry data are validated against hospital billing data every two years for the complete capture of cases and to ensure the accuracy of data for vital status at discharge.

### 2.2. Patients

A retrospective analysis of MVR or MVR + CABG from 2001 to 2017 among seven hospitals reporting to a prospectively maintained clinical registry was conducted. The analytic cohort included 1,284 patients undergoing MVR (801 bovine and 483 porcine). We excluded patients with endocarditis and those who have had a prior MVR.

### 2.3. Data Collection

Data were collected from each institution by trained data abstractors. Detailed data on demographics, past medical history, coronary anatomy and function, procedural indications and process, device type, access method, and hospital outcomes were collected using standardized reporting forms of the NNECDSG (https://www.nnecdsg.org). In 2012, hospitals began to transition their data collection tool to use the STS Adult Cardiac Surgery Database.

To ascertain survival beyond hospitalization, the NNE was linked to the National Death Index using name, date of birth, social security number, and the state vital status data. Survival data are currently updated for all patients through 2018.

### 2.4. Study Endpoints

The primary end point was all-cause mortality. Secondary end points included in-hospital morbidity, 30-day mortality, length of stay, and risk of reoperation. Reoperation was defined as any redo mitral valve replacement surgery captured by the NNE.

### 2.5. Statistical Analysis

Overall group comparisons of baseline demographics and medical histories, echocardiographic, and periprocedural and procedural characteristics were summarized using means and standard deviations, median and interquartile range, or percentages as appropriate. Differences in categorical variables were evaluated by using a *χ*^2^ test, while *t*-tests or Wilcoxon rank-sum tests were used for continuous variables.

To balance the differences in patient and disease characteristics between the two groups, a 1 : 1, nearest-neighbor propensity match without replacement was used to create comparable groups for analysis. The matching caliper was set to 0.01. The nonparsimonious logistic regression model to predict propensity scores included the following variables: age, gender, body surface area, prior PCI, prior CABG, prior stroke, preoperative atrial fibrillation, peripheral vascular disease (PVD), diabetes, chronic obstructive pulmonary disease (COPD), congestive heart failure (CHF), prior dialysis or creatinine ≥ 2, white blood cell, ejection fraction, number of diseased vessels, left main (LM) artery stenosis ≥ 50%, coronary artery disease, recent myocardial infarction (MI), and priority at the surgery. We additionally performed an inverse probability weighted (IPW) analysis to estimate the average treatment effect in the study population as a sensitivity analysis. We verified the performance of our propensity score by assessing standardized differences of the means for covariates among our propensity-matched and inverse probability-weighted cohorts [12]. Standardized differences of <0.1 indicate that the groups are comparable on a particular variable.

Survival curves showing long-term mortality were generated by the Kaplan−Meier method and compared across groups using a log-rank test. Cox proportional hazards regression was used to calculate hazard ratios (HR) with 95% confidence intervals (CIs).

Competing risk analysis, with death as the competing event, was performed for reoperation using the cumulative incidence function with the assessment of the difference between prosthetic valve types using Gray's test. Competing risk regression was used to calculate the HR and 95% CI for reoperation.

For all analyses, a two-sided *p* value <0.05 was considered to be statistically significant. Data were analyzed using the statistical software package Stata 17 (Stata Corp, College Station, TX).

## 3. Results

Overall, 1284 patients undergoing MVR with a bioprosthetic valve were identified with 801 undergoing pericardial MVR and 483 porcine MVR. The match cohort using 1 : 1 propensity score matching (PMS) had 432 patients in each group.

**Figure 1:**
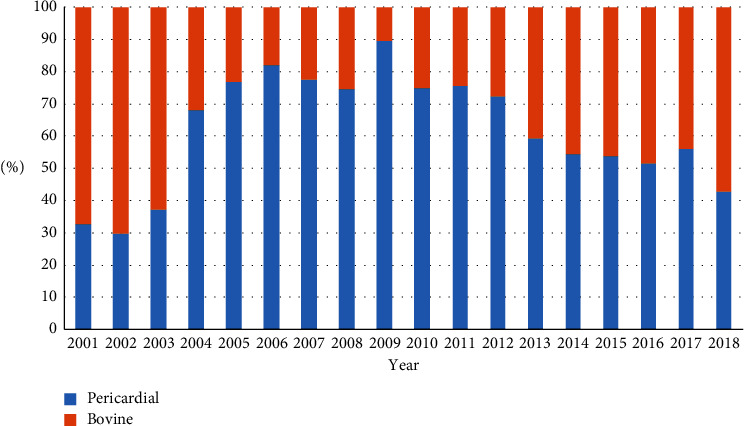
Distribution of valve type by year.

As shown in Table 1 in the overall cohort, patients receiving porcine valves were more likely to have diabetes (19.4% bovine vs. 29.0% porcine; *p* < 0.001), COPD (20.3% bovine vs. 26.7% porcine; *p* = 0.008), dialysis or creatinine >2 mg/dL (4.2% bovine vs. 7.0% porcine; *p* = 0.03), and coronary artery disease (65.3% bovine vs. 76.6% porcine; *p* < 0.001). In the propensity-matched cohorts, there were no differences in baseline patients and disease characteristics (Table 2). The distribution of valve use over the time period of our study is shown in Figure 1.

There was no difference in stroke, acute injury, mediastinitis, pneumonia, length of stay, in-hospital morbidity, or 30-day mortality in either the overall or matched cohorts (Table 3).

**Figure 2:**
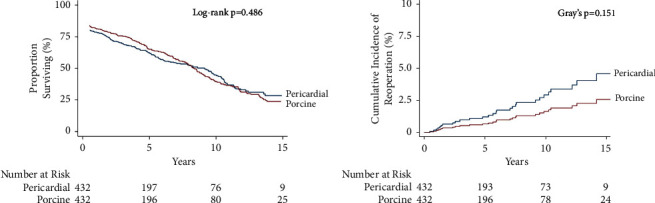
(a) Mortality and (b) cumulative incidence of reoperation in the propensity-matched cohort by tissue type.

In the unadjusted overall cohort, there was a slight significant difference in long-term survival (porcine HR 1.17 (95% CI: 1.00–1.37; *p*=0.050)) or reoperation (porcine HR 0.56 (95% CI: 0.23–1.32; *p*=0.185 (Table 4))). Long-term survival (Figure 2(a)) in the propensity-matched cohort did not differ by tissue type (log-rank = 0.486), nor did the risk of reoperation by tissue type (Gray's *p*=0.151, Figure 2(b)). This was also reflected in the HR for long-term survival (porcine HR 0.97 (95% CI: 0.81–1.17; *p*=0.756)) and risk of reoperation (porcine HR 0.54 (95% CI: 0.20–1.47; *p*=0.225)). Likewise, there were no differences in long-term survival (porcine HR 1.00 (95% CI: 0.85–1.18; *p*=>0.99)) or risk of reoperation (porcine HR 0.68 (95% CI: 0.28–1.63; *p*=0.383)) in the inverse probability-weighted (IPW) cohort.

## 4. Discussion

We found no significant difference in any of our in-hospital primary outcomes regardless of the valve type. There was a small significant difference found in long-term mortality before adjustment. While the overall incidence of reoperation was higher in subjects who underwent MVR with a bovine pericardial bioprosthetic, this did not reach significance (as shown in Figure 2(b)). Thus, there appears to be no significant difference in mortality or risk of explantation regardless of the choice of the bioprosthetic material.

There is a myriad of choices for valve replacement surgery including mechanical, cadaveric, and bioprosthetic products on the market. On one hand, with no need for lifelong anticoagulation, bioprosthetic valves are an attractive option in patients at high risk for bleeding. On the other hand, when compared with their mechanical counterparts, bioprosthetic valves are far less durable. It is important when considering a bioprosthetic valve for MVR to consider not only if but why they fail. Previous studies of bioprosthetic valves in the aortic position have shown that they are prone to fail in multiple ways, including calcification, cusp tears, and pannus formation [13]. Due to the higher closing pressure of the mitral valve, bioprosthetic valves tend to be less durable in the mitral position when compared with the aortic position. In one study of 240 patients who underwent MVR, porcine valves were shown to be more prone to failure secondary to leaflet tears, presumably due to the increased pressure. Their bovine counterparts were more susceptible to failure from adhesion of the posterior leaflet strut and leaflet to the subvalvular apparatus [14].

Studies of the hemodynamics of each valve type bring into question which valve has superior clinical outcomes and better longevity. In one study, 145 patients with rheumatic valvular disease were prospectively randomized to either bovine pericardial (Perimount) or porcine (Mosaic) valves. Pericardial valves demonstrated better hemodynamics at one year with both peak and mean pressure gradients lower in the bovine pericardial bioprosthetic valve when compared with the porcine cohort, 2 mm hg and 4 mm hg [5]. This finding suggests a plausible mechanism for the increased integrity and functionality of pericardial bioprosthetic valves. However, no difference in mortality was observed despite the differences in the two groups. This trial was limited, however, by its short-term follow-up and lack of disease heterogeneity in the patient population.

When examining long-term survival, prior reports are conflicting. Grunkemeier et al. [3] reported a lower rate of mortality within the porcine group vs. the bovine group (57% vs. 50%, respectively; *p*=0.04) in a retrospective cohort of 312 patients undergoing MVR with a bioprosthetic valve. In the same cohort, the authors also reported a greater risk of valve explantation at 15 years in those with porcine MVR when compared with their bovine pericardial counterparts (22% ± 3.7% vs. by 15 years vs. 8% ± 3.8% by 8 years). However, in a propensity-matched cohort of 802 patients who underwent either porcine or bovine pericardial MVR in a single institution, there was no statistically significant difference in survival at both 10 and 15 years between the cohorts [2]. In another single-center retrospective review of 154 pericardial MVR and 120 porcine MVR, the actuarial survival rate was calculated at 10 years with those who underwent porcine MVR 96.4 ± 0.08% vs. 94.6 ± 0.09% (*p* < 0.06) [15]. In another retrospective cohort study by Kim et al. [16] of 241 bovine pericardial MVR vs. 68 porcine MVR patients, no significant difference in short-term or long-term survival was seen. This continues the trend seen in more recent retrospective studies of overall survival. In our overall cohort, we did observe an increased mortality in the porcine group; however, these patients also were more likely to have preexisting CAD and COPD (Table 1). However, after propensity matching, our results are consistent with those of the current literature with no difference seen in survival regardless of the bioprosthetic choice suggesting that the medical comorbidities of the overall cohort may have played a role in the difference in longevity overserved.

We saw no difference in risk of reoperation in both our unmatched and matched cohorts. Again, the existing literature is conflicting regarding these outcomes. Previously reported studies have demonstrated good long-term durability of bovine pericardial valves in the mitral position such as that of Bourguignon et al. [17] who reported that the “freedom from valve-related mortality was 61.7% ± 8.9% at 20 years.” This longevity sets a high bar for long-term integrity, and early studies comparing pericardial vs. porcine MVR showed no effect of prosthesis type on the risk of structural valve deterioration [18]. Grunkemeier et al. [3] demonstrated that pericardial MVR had a lower overall risk of explantation at 10 years compared to porcine HR of 0.53 (0.34–0.81, *p*=0.003); however, when stratified to explantation for structural valve deterioration, there was no significant difference in the risk of explantation between porcine and pericardial MVR at 15 years. Interestingly, Beute et al. [2] reported a high risk of reoperation for those who underwent MVR with a bovine pericardial bioprosthetic, and the rate of reoperation for any reason was 1.89 times higher in those who underwent MVR with pericardial bioprosthetics [2]. Even more strikingly, those who underwent MVR with a pericardial valve were 2.32 times more likely to undergo reoperation due to structural valve deterioration. Furthermore, the time to reoperation was shorter for those who underwent pericardial MVR, that is, 6.8 ± 2.3 years vs. 11.1 ± 2.3 years for porcine MVR (*p* < 0.001). Similarly, Ramen et al. [15] reported a lower risk of valve explantation due to SVD, NSVD, and all-cause reoperation in the porcine vs. pericardial bioprosthesis groups. Kim et al. [16] reported results consistent with these showing no significant difference in SVD between either pericardial or porcine prosthesis. In the past, there was concern that porcine valves had an increased risk of early reoperation compared with their pericardial counterparts [14,19]. However, observational studies investigating more recent generations of pericardial and porcine bioprosthetic valves have not reproduced this pattern of reoperation [19,20].

## 5. Limitations

Our study was limited by its retrospective nature and lack of randomization. This study also suffered from different techniques and care strategies that varied between institutions and providers. However, this heterogeneity offers some benefits. Our larger sample size and variety of hospital environments may make our findings more applicable to a broader range of patients. Unfortunately, our registry did not collect anticoagulation and antiplatelet data of our patient population, and therefore, the role of these medications in this cohort cannot be ascertained with certainty. That said, there was no difference observed in bleeding (*p*=0.11) or stroke (*p*=0.44) or in the overall cohort between porcine and pericardial valves and given the lack of long-term anticoagulation with bioprosthetic valves may suggest that no difference in short-term bleeding was observed. However, further investigation is necessary before drawing any conclusions regarding the role of anticoagulant and antiplatelet use in mortality and risk of reoperation in these patients. Furthermore, there was a lack of complete echocardiographic data for all of our patients' post-MVR. Therefore, determining SVD via standardized definitions such as those proposed by the VIVID group could not be established [21]. Given this lack of echocardiographic data, our study relied on the surrogate end point of valve explant, and we were unable to track the specific causes of valve explantation. This raises the question that if SVD in one of these groups may have been a greater cause of valve failure. However, SVD is only one of the innumerable factors that may lead to the need for valve explantation. Therefore, while SVD is an important metric of long-term valve function, we believe the overall rate of explantation more accurately describes the myriad effects and durability of a given prosthetic.

## 6. Conclusion

In this multicenter analysis of patients undergoing bioprosthetic MVR, there was no difference in perioperative complications, long-term survival, or risk of reoperation between bovine pericardial and porcine prostheses. These data suggest that surgeon preference can be used to determine the best bioprosthesis for each patient.

## Figures and Tables

**Table 1:** Baseline patient and disease characteristics by tissue type.

Characteristics	Overall cohort		
	Pericardial	Porcine	P value
Number of procedures	*N* = 801	*N* = 483	
*Procedure type*			<0.001
Valve	464 (57.9%)	230 (47.6%)	
CABG/valve	337 (42.1%)	253 (52.4%)	
*Age in years, % by group*			0.087
<60 years	98 (12.2%)	38 (7.9%)	
60–69 years	197 (24.6%)	127 (26.3%)	
70–79 years	376 (46.9%)	243 (50.3%)	
>=80 years	130 (16.2%)	75 (15.5%)	
Mean	71.0 (10.0)	72.1 (8.7)	0.043
Female sex, %	385 (48.1%)	251 (52.0%)	0.18
*Body surface area, %*			0.57
<1.70	161 (20.1%)	109 (22.6%)	
1.70–1.99	356 (44.4%)	207 (42.9%)	
>=2.00	284 (35.5%)	167 (34.6%)	
Mean	1.9 (0.3)	1.9 (0.3)	0.29
Preoperative WBC >12,000 *μ*/L, %	72 (9.0%)	39 (8.1%)	0.57
Prior PCI, %	96 (12.0%)	55 (11.4%)	0.75
Prior CABG, %	41 (5.1%)	25 (5.2%)	0.96
*Comorbid disease, %*			
Vascular disease	164 (20.5%)	117 (24.2%)	0.12
Diabetes	155 (19.4%)	140 (29.0%)	<0.001
COPD	163 (20.3%)	129 (26.7%)	0.008
Atrial fibrillation	342 (42.7%)	197 (40.8%)	0.5
Congestive heart failure	446 (55.7%)	293 (60.7%)	0.08
Dialysis or creatinine ≥2 mg/dL	34 (4.2%)	34 (7.0%)	0.03
NYHA class 4	137 (17.1%)	103 (21.3%)	0.06
Prior stroke	46 (5.7%)	40 (8.3%)	0.078
*Ejection fraction, %*			0.67
<40	74 (9.2%)	47 (9.7%)	
40–49	92 (11.5%)	65 (13.5%)	
50–59	154 (19.2%)	101 (20.9%)	
≥60	380 (47.4%)	213 (44.1%)	
Missing	101 (12.6%)	57 (11.8%)	
Mean	55.9 (13.0)	55.5 (12.8)	0.68
Coronary artery disease, %	523 (65.3%)	370 (76.6%)	<0.001
Left main stenosis ≥50%	63 (7.9%)	52 (10.8%)	0.078
*Number of diseased vessels*			<0.001
1	78 (9.7%)	66 (13.7%)	
2	509 (63.5%)	257 (53.2%)	
3	100 (12.5%)	61 (12.6%)	
Valve only	114 (14.2%)	99 (20.5%)	
Myocardial infarction within 7 days	60 (7.5%)	33 (6.8%)	0.66
*Valve disease, %*			0.94
Stenosis or regurgitation	616 (79.0%)	376 (79.2%)	
Stenosis and regurgitation	164 (21.0%)	99 (20.8%)	
*Priority at surgery, %*			0.014
Elective	493 (61.5%)	260 (53.8%)	
Urgent	267 (33.3%)	200 (41.4%)	
Emergency	41 (5.1%)	23 (4.8%)	

COPD, chronic obstructive pulmonary disease; WBC, white blood cells; NYHA, New York Heart Association.

**Table 2:** Baseline patient characteristics after propensity score matching.

Characteristics	Matched cohort		
	Pericardial	Porcine	Abs. std. diff
Number of procedures	*N* = 432	*N* = 432	
*Procedure type*			0.023
Valve	207 (47.9%)	212 (49.1%)	
CABG/valve	225 (52.1%)	220 (50.9%)	
*Age in years, % by group*			
<60 years	44 (10.2%)	37 (8.6%)	0.056
60–69 years	102 (23.6%)	115 (26.6%)	0.069
70–79 years	219 (50.7%)	212 (49.1%)	0.032
≥80 years	67 (15.5%)	68 (15.7%)	0.006
Mean	71.4 (9.4)	72.0 (8.9)	0.059
Female sex, %	223 (51.6%)	224 (51.9%)	0.005
*Body surface area, %*			
<1.70	88 (20.4%)	94 (21.8%)	0.034
1.70–1.99	193 (44.7%)	185 (42.8%)	0.037
≥2.00	151 (35.0%)	153 (35.4%)	0.010
Mean	1.9 (0.2)	1.9 (0.3)	0.015
Preoperative WBC >12,000 *μ*/L, %	36 (8.3%)	35 (8.1%)	0.008
Prior PCI, %	51 (11.8%)	53 (12.3%)	0.014
Prior CABG, %	26 (6.0%)	23 (5.3%)	0.030
*Comorbid disease, %*			
Vascular disease	99 (22.9%)	104 (24.1%)	0.027
Diabetes	117 (27.1%)	116 (26.9%)	0.005
COPD	106 (24.5%)	105 (24.3%)	0.005
Atrial fibrillation	172 (39.8%)	175 (40.5%)	0.014
Congestive heart failure	268 (62.0%)	255 (59.0%)	0.062
Dialysis or creatinine ≥2 mg/dL	26 (6.0%)	23 (5.3%)	0.030
NYHA class 4	89 (20.6%)	83 (19.2%)	0.035
Prior stroke	29 (6.7%)	34 (7.9%)	0.044
*Ejection fraction, (%)*			
<40	40 (9.3%)	37 (8.6%)	0.024
40–49	63 (14.6%)	57 (13.2%)	0.040
50–59	75 (17.4%)	90 (20.8%)	0.088
≥60	207 (47.9%)	196 (45.4%)	0.051
Missing	47 (10.9%)	52 (12.0%)	0.036
Mean	55.9 (13.6)	56.1 (12.2)	0.021
Coronary artery disease, %	323 (74.8%)	323 (74.8%)	<0.001
Left main stenosis ≥50%	41 (9.5%)	43 (10.0%)	0.016
*Number of diseased vessels*			
1	55 (12.7%)	55 (12.7%)	0.005
2	237 (54.9%)	236 (54.6%)	0.021
3	53 (12.3%)	56 (13.0%)	0.012
Valve only	87 (20.1%)	85 (19.7%)	<0.001
Myocardial infarction ≤ 7 days	22 (5.1%)	32 (7.4%)	0.096
*Valve disease, %*			
Stenosis or regurgitation	332 (76.9%)	341 (78.9%)	0.050
Stenosis and regurgitation	100 (23.1%)	91 (21.1%)	0.050
*Priority at surgery, %*			
Elective	242 (56.0%)	239 (55.3%)	0.014
Urgent	170 (39.4%)	173 (40.0%)	0.014
Emergency	20 (4.6%)	20 (4.6%)	<0.001

COPD, chronic obstructive pulmonary disease; WBC, white blood cells; NYHA, New York Heart Association; ASD, absolute standard difference.

**Table 3:** In-hospital outcomes by valve tissue type.

Variables	Overall			Matched cohort		
	Pericardial	Porcine	*P*value	Pericardial	Porcine	Abs. std. diff
Number of procedures	*N* = 801	*N* = 483		*N* = 432	*N* = 432	
Return to OR for bleeding, %	52 (6.5%)	43 (8.9%)	0.11	26 (6.0%)	40 (9.3%)	0.12
Stroke, %	26 (3.2%)	12 (2.5%)	0.44	16 (3.7%)	8 (1.9%)	0.11
Acute kidney injury, %	84 (10.5%)	58 (12.0%)	0.4	50 (11.6%)	48 (11.1%)	0.01
Mediastinitis, %	9 (1.1%)	3 (0.6%)	0.36	6 (1.4%)	3 (0.7%)	0.07
Pneumonia, %	65 (8.1%)	34 (7.0%)	0.48	34 (7.9%)	29 (6.7%)	0.04
Length of stay, median (IQR) days	8.0 (6.0–13.0)	8.0 (6.0–14.0)	0.32	9.0 (6.0–13.0)	8.0 (6.0–13.5)	0.04
In-hospital mortality	92 (11.5%)	60 (12.4%)	0.61	59 (13.7%)	50 (11.6%)	0.06
30-day mortality	98 (12.2%)	61 (12.6%)	0.84	61 (14.1%)	51 (11.8%)	0.07

**Table 4:** Crude and adjusted hazard ratios for long-term outcomes.

Models	Tissue type	HR (95% CI)	P value
*Mortality*			
Unadjusted	Pericardial	1.00 (ref)	
	Porcine	1.17 (1.00–1.37)	0.050

PSM	Pericardial	1.00 (ref)	
	Porcine	0.97 (0.81–1.17)	0.756

IPW	Pericardial	1.00 (ref)	
	Porcine	1.00 (0.85–1.18)	>0.99

*Reoperation*			
Unadjusted	Pericardial	1.00 (ref)	
	Porcine	0.56 (0.23–1.32)	0.185

PSM	Pericardial	1.00 (ref)	
	Porcine	0.54 (0.20–1.47)	0.225

IPW	Pericardial	1.00 (ref)	
	Porcine	0.68 (0.28–1.63)	0.383

PSM, propensity score match; IPW, inverse propensity weighting.

## Data Availability

The data that support the findings of this study are available from the corresponding author upon request.
